# Divergent migratory strategies lead to variable refueling performance amongst Gray Catbirds *(Dumetella carolinensis)* during spring stopover in the Gulf of Mexico

**DOI:** 10.1186/s40462-024-00518-1

**Published:** 2025-10-15

**Authors:** Michael Griego, Mariamar Gutierrez Ramirez, Alexander R. Gerson

**Affiliations:** 1 Organismic and Evolutionary Biology Program, University of Massachusetts Amherst, Amherst, MA 01003 USA; 2 Department of Biology, University of Massachusetts Amherst, Amherst, MA 01003 USA

**Keywords:** Migratory stopover, Energetics, Body composition

## Abstract

Each spring, migratory Gray Catbirds (*Dumetella carolinensis*, henceforth referred to as Catbirds) that have wintered in Central America and the Caribbean, fly across the Gulf of Mexico during their northern migration. These long-distance flights are primarily fueled by lipid reserves, but protein is also catabolized leading to significant depletions in organ and muscle tissue mass upon arrival at stopover sites. Here, Catbirds must not only recover from their previous intensive flight across the Gulf but must also provision for the next leg of migration as they continue to their breeding grounds in North America. There is still much to be known about how reduced lean mass at stopover may constrain refueling performance. Catbirds present a unique opportunity to study refueling performance relating to differential migratory strategies as these songbirds have significant geographic spread in their North American breeding grounds. We caught Catbirds during spring migration on St. George Island, FL over three consecutive years. We measured total fat and lean mass using non-invasive quantitative magnetic resonance, basal metabolic rate using standard flow-through respirometry techniques, and plasma triglycerides to determine refueling rate. We then used feather deuterium (δD) to estimate breeding location and migratory distance. We determined, with high probability, three migratory strategies used by this group of Catbirds at stopover, yet found no differences in total, fat, or lean body mass amongst birds attributed to be short, medium, or long-distance migrants. However, migratory distance explains the relationship between total mass and refueling rates, with long-distance migrants having a larger increase in plasma triglyceride concentration per unit increase of body mass compared to short-distance migrants. Furthermore, we document a physiological strategy whereby long-distance migrants can deposit greater amounts of lean and fat mass with a smaller associated increase of metabolic rate than predicted for their size.

**Supplementary Information:**

The online version contains supplementary material available at 10.1186/s40462-024-00518-1.

## Introduction

Migratory stopover is a critical period in songbird life history that provides the animal with resources to recover from a previous flight and allows them to stage for the remainder of their journey. To meet the energetic demands of migration, birds use lipids as a primary fuel, while also burning significant amounts of endogenous protein [21, 35]. Although the costs and benefits of catabolizing protein remain unclear, it has been hypothesized that this strategy may result in reduced metabolism upon arrival and thereby decrease maintenance costs during stopover refueling [22, 36]; [20, 25]. It is has been clearly demonstrated, however, that burning protein during flight leads to dramatic reductions in organ sizes and total lean mass which must be replenished quickly during migratory stopover refueling [3, 5, 6, 38]. Such reductions of mass during flight may result in lethal or sublethal physiological deficits during stopover refueling and can constrain refueling performance and thus require longer durations at stopover. Ultimately, extending the time spent refueling during migratory stopover could have many negative implications for birds as they make their journey to breeding grounds (Seewagen et al., 2013). Gutierrez Ramirez and colleagues [29] show that stopover duration decreased by 22% in Northern Waterthrush for each additional gram of lean mass deposited during migratory refueling, which demonstrates a connection between time-optimization and body condition at stopover.

In many species of migratory songbird, early arrival on the breeding grounds correlates strongly with increased clutch size, higher quality mates, and higher nestling survivorship [30, 40, 45, 47]. Thus, traits optimized for rapid refueling at stopover impact individual survivorship and have important implications at the population-level. Therefore, investigations into physiological flexibility with regard to whole-animal performance at stopover can add greatly to the understanding of songbird ecology. The degree to which certain physiological, behavioral, and anatomical traits increase flight and refueling performance can be clearly contrasted between facultative and obligate migrants, and non-migratory species [41, 51, 59, 61, 68]. Less certain, however, is how beneficial phenotypic variation might be within a single species which exhibits multiple migratory strategies. Though lean body mass is the metabolic tissue that underpins whole-animal performance, it is still not well-documented how body condition is related to refueling performance within species that have evolved divergent migratory strategies [1, 23, 50, 62, 63]. For example, songbird populations whose migration spans the entirety of North America may exhibit differential physiological and behavioral strategies related to optimal refueling when compared to their conspecific relatives that breed in the southern US.

Neotropic-nearctic migrants experience different environmental and ecological pressures as they move from their wintering grounds to breeding locations. As such, they rely on phenotypic flexibility to sustain maximal performance in unpredictable situations [24, 58, 59, 68]. Flexibility with respect to metabolism may allow migratory birds to adjust metabolic demands to the demands of the current life history stage. For example, it has been documented that migratory land and seabirds reduce metabolic rates after long-duration flights [4, 20], which may decrease night time thermoregulatory costs, allowing greater multi-day fat accumulation. Reductions in metabolic rate have been documented in association with in-flight catabolism of fat and lean body tissues [25, 39, 56]. Recent evidence also suggests that songbirds increase flight muscle mass, and metabolic rates in anticipation of migratory flight [15], which has been thought to support extended duration flight by increasing both flight muscle strength and energetic capacity, respectively [5, 12, 65]. Despite advancement documenting the relationship between metabolism and lean muscle mass, the implication for migrants’ refueling performance at stopover grounds remain minimally explored. Of particular importance is understanding the interplay of metabolism, refueling performance, and the deposition of both lean and fat mass as they stage for the next leg of their journey.

The Catbird is an ideal species to investigate the interplay of lean body mass, metabolism, and refueling performance in songbirds at stopover as they stage for the next leg of their flight. Though widespread across North America during the breeding season, Catbirds have a wintering range in Central America, the Caribbean, and in some areas of the U.S. Gulf Coast. Individuals that differ significantly with respect to remaining migratory distance to breeding grounds may have overlapping distributions in some stopover habitats on the U.S. Gulf Coast during the spring, which allows for comparisons among migratory strategies while sampling at single site [60]. Moreover, Marsh (1983) has shown that Catbirds initiate seasonal hypertrophy of flight muscles to facilitate migratory flight performance [48]. We selected St. George Island, FL USA as study system as it presents the unique opportunity to sample birds immediately after completing a trans-Gulf flight from their wintering grounds in the Caribbean and Central America during spring migration and investigate refueling performance in preparation for the next leg of their migration. Lastly, because migrants using this island for stopover breed at vastly different latitudes, we can isolate the effects that migratory strategy may have on body mass deposition and metabolism during stopover. By sampling at a common stopover site, we control for differences in refueling performance that might be an artifact of variable habitat quality when sampling at multiple sites: the individuals in this study are temporally synched and experience similar ecological and environmental factors during stopover on the island. We can therefore attribute differences in refueling performance relative to breeding destination.

This study primarily focuses on the interplay of body composition with metabolic and refueling rates in migrants. For a refueling bird, metabolic and refueling rates act as contrasting forces during stopover. Typically, refueling birds forage vigorously during the day to replenish fat and lean tissues, but during the night they fast and must draw against these endogenous reserves to sustain their metabolism. Because Catbirds are known to have high breeding site fidelity, we were able to use feather hydrogen isotopes to determine *previous* year breeding locations of our focal animals and thus distinguish each individual as either short, medium, or long-distance migrants (Fritze & Suthers, 2005). We then related metabolic performance during stopover to these differing migratory strategies. We hypothesized that migratory birds would have a phenotype that maximizes performance in the context of their respective migratory strategy (long vs. short-distance), and long-distance migrants would have greater stopover refueling performance in comparison to their short-distance conspecifics. We anticipated that long-distance migrants would have larger deposits of fat and muscle in preparation for their upcoming flight, compared to short-distance migrants. Furthermore, we tested whether basal metabolic rates (BMR) are influenced by an individual’s total lean mass during stopover refueling. We expected that birds at stopover in poor body condition (i.e., with less lean mass as a result of their previous flight across the Gulf) will have lower metabolic rates [4, 20, 25, 39, 58]. We expected to see a relationship between metabolism and body condition over the course of refueling; as birds redeposit lean mass, they will in turn increase their metabolic rates, proportionally. However, due to increased fat, lean, and body mass resulting from such fuel deposits, we also expected to see higher metabolic rates in long-distance Catbirds.

## Methods

### Study site and focal species

**Fig. 1:**
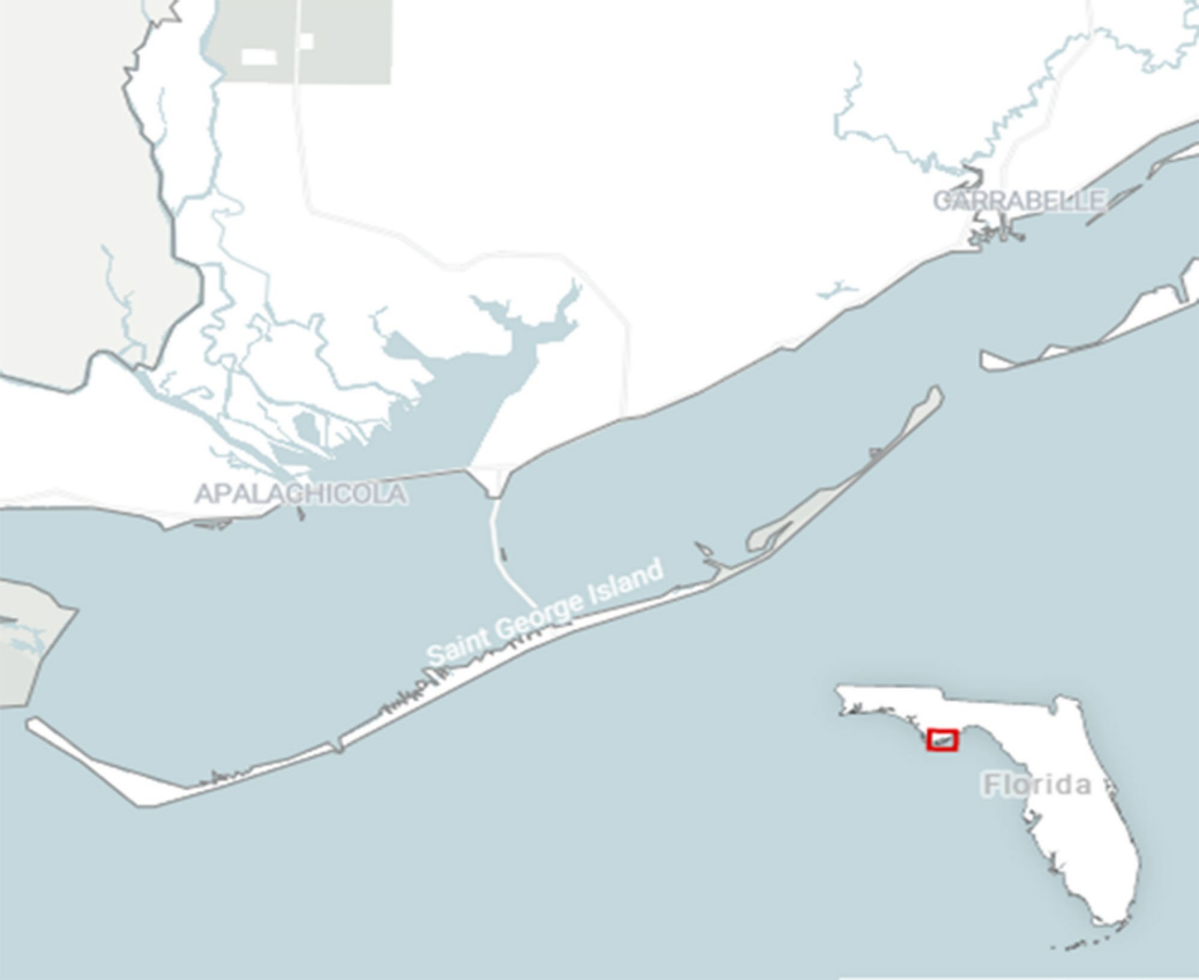
St. George Island, FL USA is a narrow barrier island characterized by a wide variety of habitat types and is essential refugia for migratory songbirds immediately after crossing the Gulf of Mexico

Data were collected on St. George Island, a barrier island in the Gulf of Mexico off the panhandle of Florida, during the spring seasons of 2016–2018 (Fig. 1). Like many barrier islands off of the U.S. Gulf Coast, St. George Island is the first chance for landfall for many trans-gulf migrants. At 33 km long with a variety of habitat types including palmetto scrublands, marshes, dunes, woodland grasses, and mature pine and live oak stands (Lester et al. 2016; Edmiston, 2008), St. George Island provides necessary stopover habitat for spring migrants, and Catbirds have been documented to stay at this site as long as 21 days after arrival [16]. These habitats are essential not only for assisting recovery from the previous trans-Gulf flight, but also provide the opportunity for birds to deposit large amounts of fuel in preparation for continuing migration. Catbirds are numerous on the island during spring migration, with individuals wintering from southern Florida to Central America and the Yucatán peninsula. Notably, some migrants passing through St. George Island can continue for up to 1,500 miles to reach their breeding grounds in Canada. Furthermore, migratory Catbirds have been observed to significantly adjust body mass in preparation for long distance flight, which make them an ideal species to investigate the relationship between body condition and whole-animal performance in the context of migration and stopover refueling [15, 48]. Sampling here gives us a unique ability to measure whole-animal performance during a brief phase where birds must both recover from a previous flight, and simultaneously prepare for the next leg of their migratory journey.

### Capture and sampling

Birds were captured using standard mist netting techniques during three consecutive spring seasons (2016–2018) at Unit 4 of the Apalachicola National Estuarine Research Reserve on St. George Island, FL USA. Net hours typically spanned from 0630 to 1300, and 1700–1900 daily, from 01 April to 12 May. Body mass, morphometrics, and age (Pyle, 1997) were recorded at the time of banding (USGS permit # 23979). Unflattened wing chord and tail length were measured using a wing ruler (to 1 mm) and tarsus, nares to tip, exposed culmen, bill width, and sternum length were measured using digital calipers (to the 0.01 mm). Fat deposits were visually scored on a 9-point scale [37] and pectoral muscle was scored on a 4-point scale [2]. Body mass was measured with a digital balance (to the 0.01 g; Ohaus, Scout Pro SP402, Parsippany, New Jersey, USA). The right third rectrix (R3) was collected for later analysis of deuterium (δD), as tail feathers are grown at the breeding site and can be used to determine individuals’ locations during the previous summer [9]. No individuals were observed actively replacing tail feathers.

To measure plasma triglyceride concentration, blood was sampled via brachial puncture within 20 min of capture using a 26-gauge needle (BD Medical PrecisionGlide) and capillary tube and was samples were < 1% of estimated blood volume (Fair et al., 2010). Blood samples were centrifuged no later than 13:00 each day, at 1000 rpm for 10 min to separate plasma from red blood cells. Plasma was carefully separated using a pipette and transferred to a cryogenic vial and stored in liquid nitrogen for the remainder of the field season. Upon return to the lab, plasma samples were transferred to a -80 °C freezer until assays were performed. Birds were held for no more than 24 h to collect metabolic data using standard flow-through respirometry as permitted federally (USFWS: MB65968B-0) and by UMass IACUC (#2015-0019) and Florida state permit (#23979). Immediately prior to metabolic testing, all birds were scanned using Echo MRI Technology Quantitative Magnetic Resonance (QMR) to accurately determine total fat and lean mass of the individual without the use of invasive procedures [27]. Individuals that had very low body fat, or otherwise appeared to be stressed were released at least 2 h before sundown and were not included in metabolic testing.

### Stable isotope analysis

The use of feather deuterium (δD) isotope analysis is a powerful tool that can be used to determine breeding locations of many species of birds caught outside of their summering grounds if they molt at their breeding grounds [9]. Deuterium, a stable isotope of hydrogen, is incorporated through the animal’s diet and is assimilated into feather tissues where it remains biologically inert. Many songbirds, including the Catbird, only molt their tail feather at their breeding grounds, thus incorporating the local hydrogen isotope ratio (deuterium relative to monoprotonated hydrogen) into the feather tissue [19]. Under laboratory analysis, the local δD signal present in the feathers can then be matched to geographic regions using an empirically determined isoscape of North America. Furthermore, because Catbirds exhibit very high breeding-site fidelity, we can reliably estimate distance remaining to breeding grounds when capturing actively migrating birds during northward spring migration using this method. Individuals selected for feather δD analysis were a subset of all Catbirds caught in the field, and had completed metabolic trials, plasma metabolite analysis, and two body composition scans using quantitative magnetic resonance (detailed below under *Metabolic Rates and Body Composition*). For laboratory isotope analysis, we prepared feather samples (*n* = 42) in house at the University of Massachusetts Amherst (Amherst, Massachusetts, USA) using a 2:1 chloroform: methanol wash to remove surface lipids and other impurities which can interfere with mass spectrometry similar to Hobson [32]. Following the initial wash they were rinsed in clean C: M solution an additional four times as recommended by Paritte & Kelly [53], and allowed to air dry for two days while stored in clean envelopes. The dried feather samples were sectioned into approximately 0.2 mg pieces and sealed into 3 × 5 mm silver capsules and allowed to equilibrate to local water vapor δD for three weeks before analysis at the University of New Mexico Center for Stable Isotope Research. Using a high-temperature conversion elemental analyzer (Thermo- Finnigan TCEA) in-line with an isotope ratio mass spectrometer (Thermo-Finnigan Delta Plus XL), δD values were measured for each sample and were expressed in values of δ derived from the following equation as:

$$\delta \text{D}=1000^*\,\text{[(Rsample - Rstandard/Rstandard)}$$

**Fig. 2:**
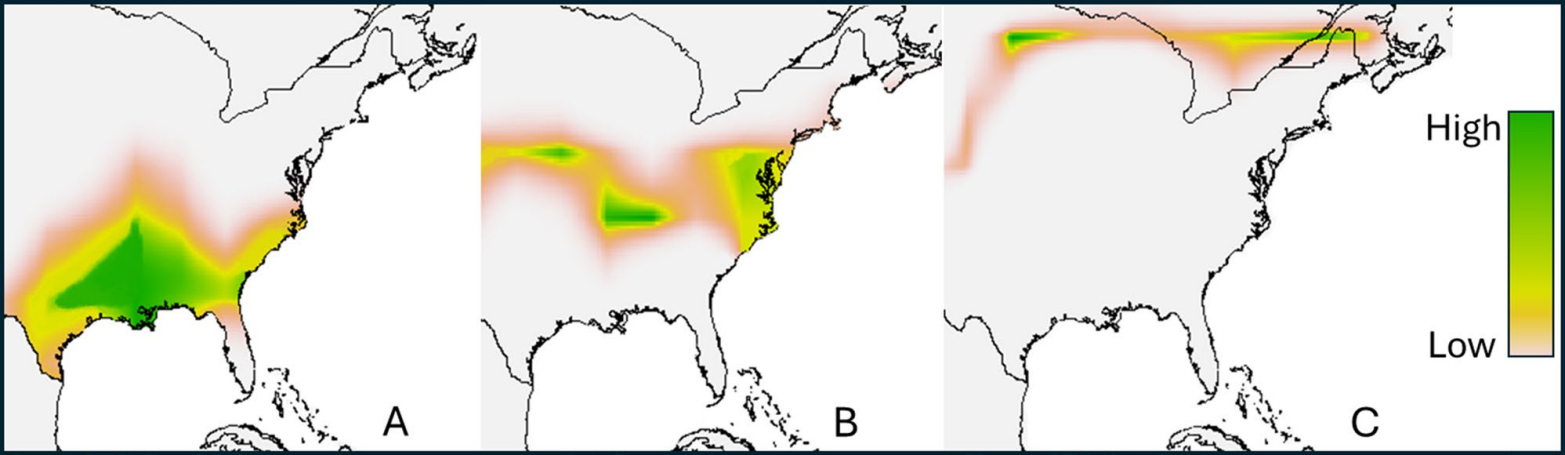
Probable breeding distributions of focal Catbirds in North America. Tail feather deuterium (δD) was used to separate all birds (*n* = 42) into three migratory strategies: short-distance (**A**; *n* = 10), medium- distance (**B**; *n* = 12), and long-distance migrants (**C**; *n* = 20). The darker green shading has the highest probability that all individuals within the given migratory designation originated from that locality. Long-distance migrants had an estimated 1,400mi journey to complete after stopover at St. George Island

The units presented in the results are expressed as parts per mil (‰). Three laboratory keratin references were used as comparative standards (δD: −47‰, − 54‰, − 93‰, − 174‰), and δD variation (SD) of these was less than 4‰ during analysis. Then, using the feather isotope data, we constructed a δD isoscape using *assignR* package [46] in *R* software (Fig. 1). The isoscape is constructed using known environmental precipitation δD values for North America and the Caribbean. We then calibrated our sample δD to birds with known breeding locations using data from (Hobson, Van Wilgenburg, et al., [34]) native to package assignR. We constructed posterior density maps to estimate the geographic breeding range for each individual, selecting for the highest 10% probability of range. Individuals that had both refueling and metabolic data in addition to isotopes were then binned into short (*n* = 10), medium (*n* = 12), or long-distance (*n* = 20) migratory groups based on 90% probability that every member within the group originated in the assigned range (Fig. 2). Thus, remaining distance to breeding grounds after capture is approximately < 120mi, 400 to 700mi, and 900 to 1,200mi for the respective groups. We opted against an analysis that treats δD as a continuous variable chiefly due to limitations inherent to the isotopic geography of our study system. While it is true that δD can be a reliable (albeit coarse) proxy for latitude when looking at continental-level spatial scales across North America, there are some important caveats when using this tool in the gulf system. It has been known for some time that δD is highly variable in the Gulf of Mexico (GoM) and surrounding coastal land mass [18] This is due to the mixing of water from equatorial ocean currents with incurrent continental water from rivers. Further muddling this is that intense storms characteristic to the Gulf of Mexico have the ability to send this mixed water back inland in the form of precipitation. As a result, organisms that uptake water as far north as Georgia, can have tissue δD values that are within analytical error of animals in the Yucatan peninsula, some 800 miles south. This has been recorded in multiple species, including insects (Hobson, Soto, et al., [33]). Thus, the relationship between deuterium and latitude when focusing on *less* depleted δD values (i.e. coastal values) is non-linear. We therefore cannot assume δD to be a reliable linear proxy for latitude when constructing models for highly mobile animals near the gulf coast.

### Metabolic rates and body composition

After initial capture and prior to respirometry, birds were held with ad lib access to food (meal worms, Mazuri ground diet, and dehydrated berry mixture) and water, and were monitored for health. All birds (*n* = 61) began respirometry on the evening of their capture and were at least 2 h post-absorptive upon the start of data collection. Immediately prior to being put into respirometry, birds were rapidly scanned using an Echo MRI QMR to assess total lean and fat mass. Metabolic rates were measured overnight in a thermal-neutral environmental chamber (T_a_= 30 °C) from 20:30 to 06:00. Once finished with BMR measurements the following morning, birds were monitored for health, scanned in the QMR for post-respirometry body composition, and released back into stopover habitat at the field site where they were caught. Basal metabolic rate (BMR) measurements were taken on up to 6 birds per night with the use of rapid multiplexing. Excurrent CO_2_ and H_2_O were measured using a LI-840 A (Licor, Lincoln NB USA), and O_2_ was measured using an FC-10 oxygen analyzer (Sable Systems, Las Vegas NV USA). Data were collected using Expedata software (Sable Systems, Las Vegas NV USA). $\dot{V}O_2$ and $\dot{V}CO_2$ and $\dot{V}H_2O$ were calculated using standard equations for push respirometry found in Lighton (2008). We present metabolic rate expressed as terms of milliliters of oxygen consumed per minute. $\dot{V}CO_2$ measurements are taken alongside $\dot{V}O_2$ to also calculate respiratory quotient (RQ), the proportion of carbon dioxide produced to oxygen consumed by the animal. RQ is an important check to ensure that animals are postabsorbtive, an important condition when measuring basal metabolic rates. We define BMR as the mean of the lowest 2 min of $\dot{V}O_2$ during the overnight run. We also present a log-log linear model of body mass to BMR in our analysis as these relationships are of importance to the understanding of intraspecific allometric scaling principles.

### Refueling rates

Plasma metabolites, particularly triglyceride concentration [TRIG] can be a reliable indicator of refueling rates in migratory songbirds, and we performed metabolite assays as in Guglielmo and colleagues [26]. Samples were diluted three-fold with 0.9% NaCl solution. Using a standard triglyceride and glycerol kit (Sigma Aldrich F6428 and T2449, respectively), assays were performed using a microplate spectrophotometer (BioTek Synergy H, VT, USA). Each sample (*n* = 44) was run in duplicate, in 96-well microplates and read at 540 nm. Concentrations of triglycerides were determined via comparison to a standard curve (standard concentrations provided by the Sigma Aldrich kits). All data included in analyses were from samples with coefficients of variation (CV) values less than 15%.

### Data analysis

The relationships between basal metabolic rate ($\dot{V}O_2$in mL min^− 1^) and body composition (expressed in terms of total, lean, and fat masses) of Catbirds were investigated using linear modeling. We attempted to account for body size by standardizing to linear measurements using unflattened wing chord and tarsus lengths (as in Peig & Green [54]), however these were not found to be significantly correlated to size. Additionally, age of bird, and year of capture were not found to be significant in our models and are not included in the statistical summaries. We compared body composition and refueling rates across migratory groups using ANOVA (α = 0.05) with pairwise-comparisons reported where relevant, using Tukey’s HSD test. Plasma triglyceride concentrations were log_10_ transformed to normalize data as in Guglielmo and colleagues [28]. All statistical tests and plots were created in R version 4.1.3, using packages NLME, ggplot2, and assignR. Breeding location was determined using the package assignR as in [10]. Metabolic scaling coefficients are calculated by the slope of a log-transformed model of metabolic rate regressed against log-transformed body mass in grams.  

## Results

### Body composition

**Fig. 3:**
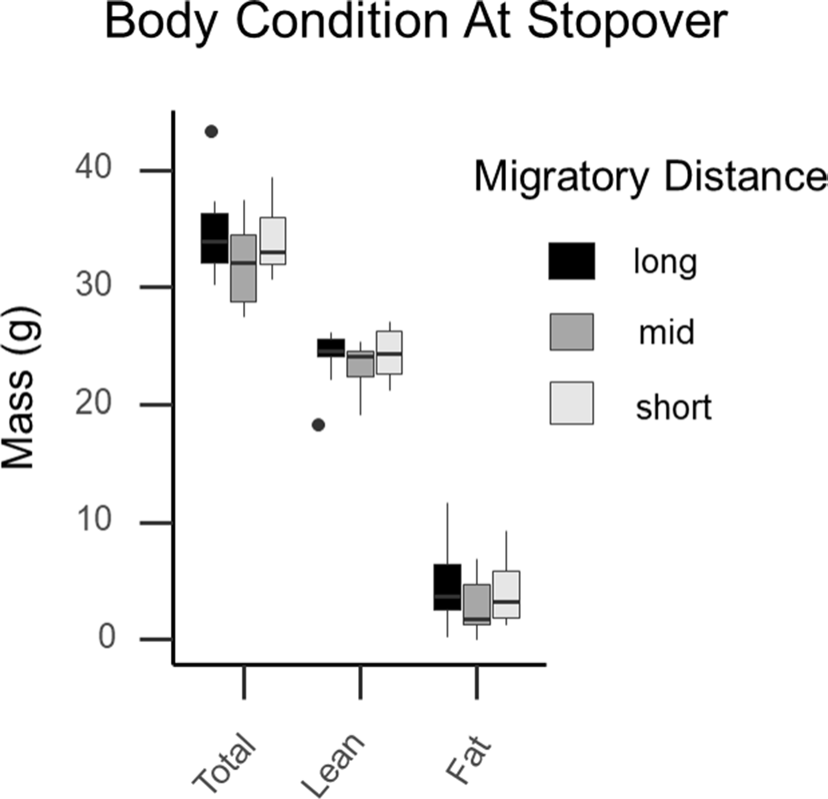
Catbird body composition at stopover. No significant differences were documented with respect to total, lean, or fat mass in Catbirds of varying migratory strategies caught during active refueling

Average body mass was 33.50 g for all birds, and did not significantly vary among long, medium, or short-distance migrants (F_(2,36)_ = 1.4231, *P* = 0.2542; Fig. 3). Total fat mass averaged 4.10 ± 2.9 g (8% of body mass) for all birds and similarly did not vary among migratory groups (F_(2,36)_ = 0.600, *P* = 0.554). Lean mass averaged 24.78 ± 1.79 g, and a Tukey HSD revealed a near significant difference between long-distance and middle- distance groups (F_(2,36)_ = 2.124, *P* = 0.114). Neither total mass, fat mass, nor lean mass correlated with any linear morphometric which we collected (tarsus, wing cord, bill length) and so no size corrections were performed on our data, as we found no differences in linear measurements and migratory distance.

### Refueling rates

**Fig. 4:**
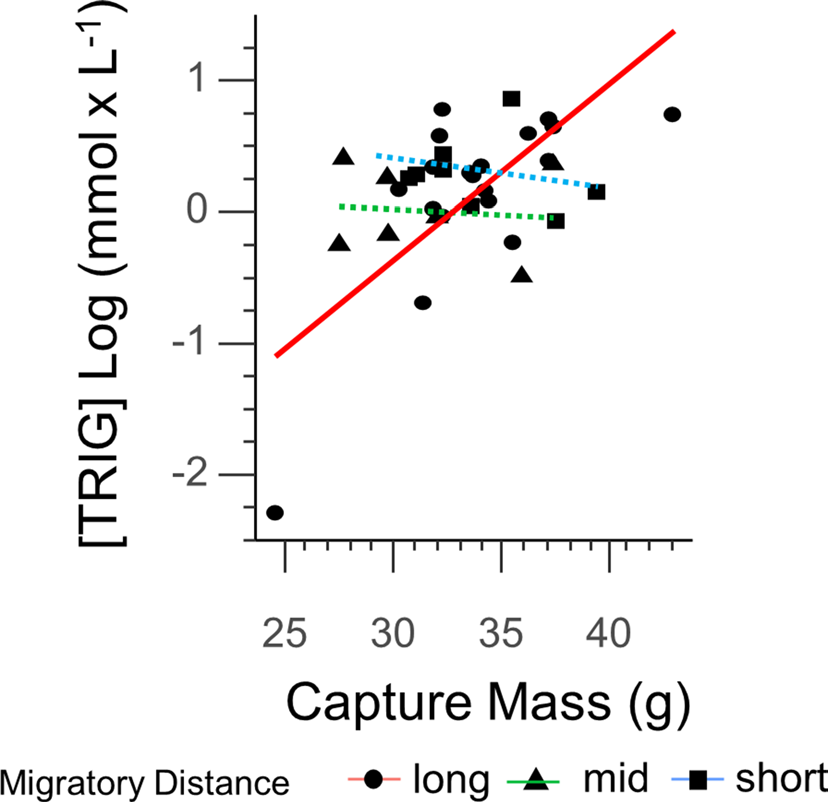
Refueling rates as measured by plasma [TRIG] significantly correlated to total body mass in long-distance migrants (solid red line). No significant relationship was present in medium or short-distance migrants (dashed green and dashed blue lines, respectively)

**Fig. 5:**
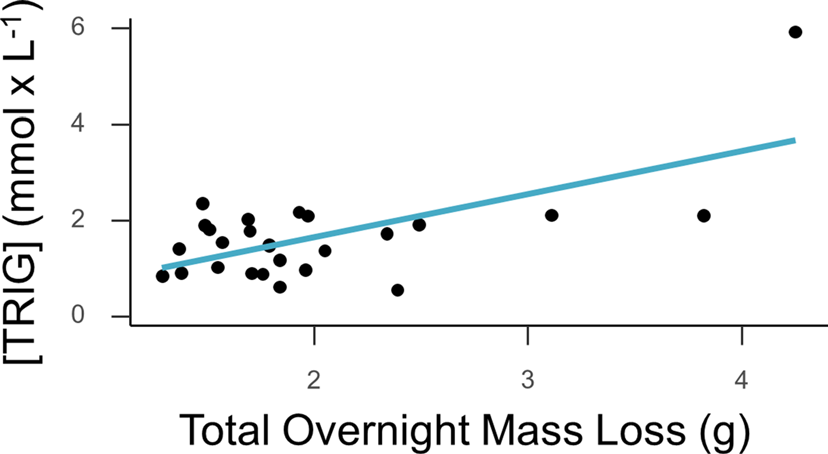
A subset of birds (*n* = 27) were measured for overnight mass loss during respirometry. We found evidence that high rates of overnight mass loss in association with birds having higher rates of refueling

The average [TRIG] values for all birds measured was 1.41 mmol/L and did not vary significantly between long, medium, or short-distance migrants (F_(2,36)_ = 1.062, *P* = 0.370). For [TRIG] there was a significant interaction between body size and migration distance (F_(2,36)_ = 9.821, *P* = 0.003; β = 0.05; std error = 0.4247), so we further analyzed this relationship within each migratory group. There was only a significant and positive relationship between body mass and refueling rate in long distance migrants (F_(5,32)_ = 17.08, *P* = 0.0029; Fig. 4) but no similar relationship was found in short or medium distance migrants (*P* > 0.05). Removing the individual with the lowest mass from the long-distance group did not change the significance of the relationship (F_(5,31)_ = 2.55, *P* = 0.04). A pairwise contrast showed significant differences in the slope between long and medium-distance migrants (*P* = 0.01) and long and short-distance (*P* = 0.02), but no difference between medium and short-distance migrants (*P* > 0.05). Across all groups, fat mass was related to TRIG (F_(2,36)_ = 12.57, *P* = 0.001; β = 0.08; std error = 0.4125). Lean mass was not found to be significantly related to TRIG (*P* > 0.05). In birds where overnight mass losses could be calculated (*n* = 27), TRIG at capture was positively related to greater overnight total mass loss (F_(1,26)_ = 16.796, *P* = 0.004; Fig. 5). Removing the individual with the highest overnight mass loss yielded a near-significant relationship (F_(1,25)_ = 0.4016, *P* = 0.053).

### Basal metabolic rates

**Fig. 6:**
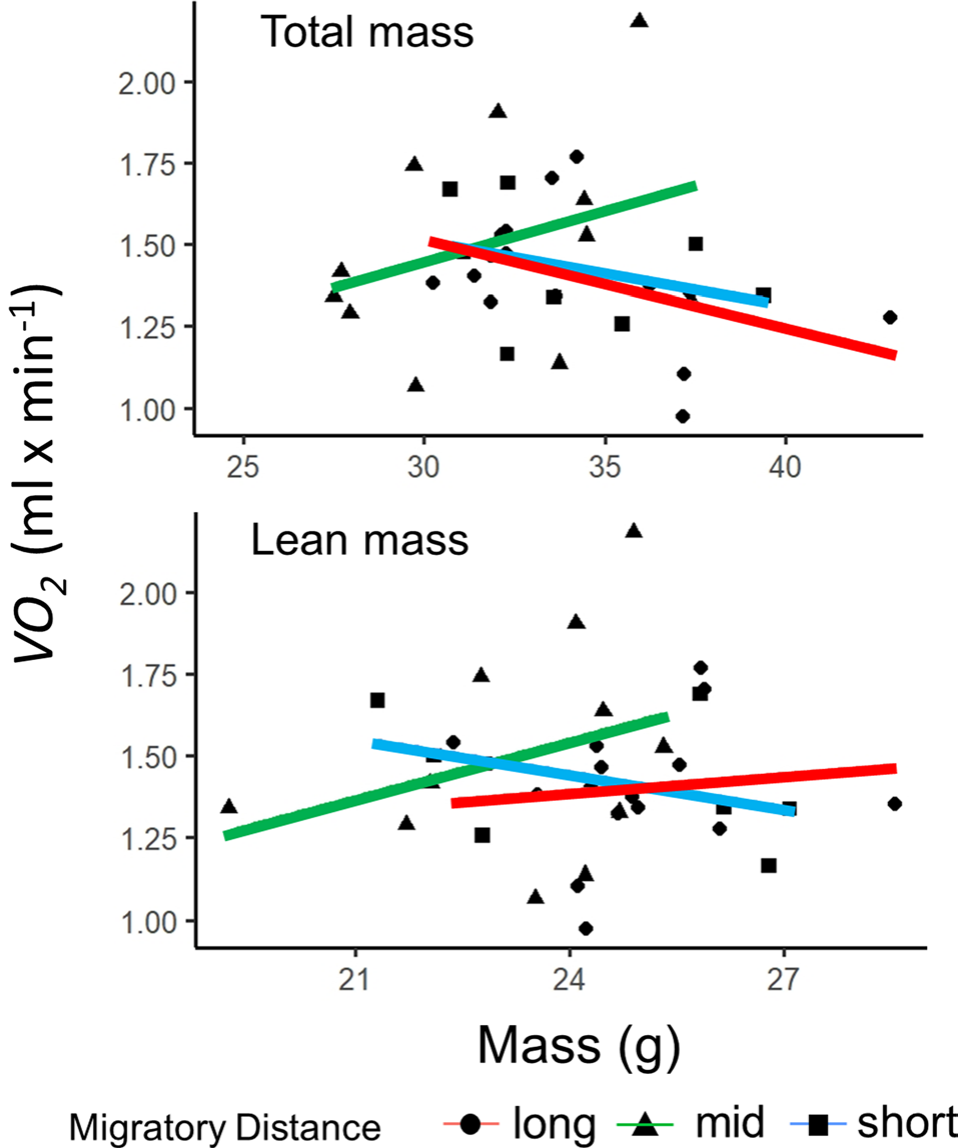
BMR was not associated with total or lean mass as predicted by allometric scaling laws. In long-distance birds, we measured a negative association of BMR and total mass, indicating that these birds employ a physiological strategy to save energy even with large body mass

Basal metabolic rates ($\dot{V}O_2$) averaged 1.36 ml min^− 1^ (*n* = 61), with a mean RQ of 0.71. We found no differences in metabolic rates between short, medium, and long-distance migrants (F_(2,36)_ = 1.0262, *P* = 0.372; Fig. 6). When considering the slope of the log-log transformation of BMR and total body mass, we present short-distance migrants as having a scaling coefficient of 0.13, middle-distance migrants as 0.61, and long-distance migrants having a scaling coefficient of -0.35. Notably, BMR did not account for overnight mass losses across all birds measured, as there were no associated increases in $\dot{V}O_2$ with increased mass loss (F_(1,34)_ = 0.8989, *P* = 0.350).

## Discussion

Our study of within-species variation in stopover performance provides greater understanding of how songbirds simultaneously recover from long-distance flight and provision for the next leg of their migration during migratory stopover. This study design is the first to investigate how lean mass governs refueling performance and metabolism, and relate those interactions to migratory distance using stable isotope to estimate breeding locations. We show evidence that Catbirds’ refueling rates scale positively with total body mass within individuals furthest from their breeding grounds, while simultaneously incurring little metabolic penalty for greater mass during migratory stopover refueling. We also present evidence that refueling rates are significantly related to overnight mass loss across all individuals at stopover and is the first documentation of such a relationship to our knowledge. Our approach provides the critical analysis of how songbirds make phenotypic adjustments during migratory stopover in anticipation of their next flight in as little as a few days. In combination with the stable isotope data, we provide important distinctions as to the physiological strategies birds employ during this essential life history event. We show that even within a species or population, challenges are not uniform and neither are the ways that animals cope. We hope that these first attempts to delineate differential physiological strategies within species during migration encourage scientists to use these tools and reexamine previously studied systems.

Though we cannot account for differential challenges faced by individual birds during their prior flight, by sampling at a single site, we ensure that all birds were subject to similar environmental conditions, ecological pressures, and refueling habitat quality at stopover. We can, therefore, draw inference that the documented refueling strategies are likely being made in anticipation for the next leg of the journey, and not solely as a consequence of the previous trans-gulf flight. We found that short-distance migrants do not significantly increase their refueling rates proportionally to mass gain. This is in congruence with previous findings on biphasic mass recovery during stopover in which birds initially deposit lean mass prior to replenishing lipid reserves [8, 24]. It may be such that this group of birds has sufficiently replenished lean tissue mass and regained optimal digestive performance without further need to deposit large amounts of fat mass, as their breeding grounds may be as little as a few miles away on the mainland. Griego et al., [24] shows that passerines can overcompensate digestive tissue mass and function within as little as 48 h of stopover refueling, though a potential tradeoff is the increased metabolic costs associated with maintaining large digestive systems. Building large organs, and incurring the associated metabolic cost, solely to deposit large fat reserves would be of no benefit to the short-distance migrants who only have a small distance left to cover. To increase migratory pace, however, long-distance migrants would benefit from rapidly depositing as much fat as possible during their first stopover and thereby reduce the total number of stops en route to breeding grounds. In this scenario, digestive organ tissue would be a necessity regardless of the metabolic cost. This potentially explains why we found higher refueling rates associated with greater body mass in long-distance migrants, and not within short-distance individuals.

Having high breeding site fidelity and a vast geographical distribution in North America, Catbirds are an ideal species when considering the use of feather δD to estimate breeding range. Even when sampled during active migration many hundreds of miles from their breeding grounds, we can estimate the distance left on their journey in relation to where they stop to refuel with reasonable confidence. Our sampling included individuals that vary widely in migration distance, yet we found no differences in body condition across short, medium, or long-distance migrants similar to Hays et al. [31]. This result may not be surprising, as birds in this study completed a trans-gulf flight within only a few previous days of capture and were likely in various stages of refueling. Without the use of tracking technology, it is impossible to say with certainty how long birds have been on the island, and therefore body mass data cannot be standardized by number of days refueling. Yet with the use of the emerging stable isotope analyses and QMR, we have identified novel metabolic strategies which may help migrants cope with the demands of migration.

This research documents a great breadth of physiological performance, particularly with respect to how high rates of daily refueling are associated with high rates of overnight mass loss. To our knowledge, this is the first documentation of such a finding and provides a new perspective on the consequences of metabolism on body condition during stopover. In our experience with Catbirds and other migratory species on the island, high rates of overnight mass loss are frequently documented. Notably, the individual with the highest overnight mass loss catabolized their fuel reserves in roughly a 3:1 lean mass to fat ratio. It is within reason to speculate that this individual was very near the end of stopover refueling and was ready to depart; having large digestive tissue enabled high rates of refueling during the day and provided a fuel source overnight so that it could spare vital fat reserves. This finding fits with what has been observed with biphasic refueling, and lean mass dynamics immediately prior to flight. We strongly emphasize the inclusion of all data points in our analyses, and caution against culling observations that may at first seem extreme. For example, in Fig. 4 we document an individual with low capture mass relative to others in the study. However, in our experience, this low mass is not atypical for a Catbird that has just recently crossed the Gulf of Mexico and its inclusion did not affect statistical analysis. We have high confidence in the analytical precision of our instrumentation and have no reason to believe that such a range of values are a consequence of measurement or statistical error. As such, we present the full scope of physiological performance observed in refueling Catbirds to better inform hypotheses regarding the extent by which flexible phenotypes may assist animals during challenging life history events. In the case of songbirds, it has been hypothesized that the migratory phenotype has evolved in a large part to support the energetic needs required to complete intensive intercontinental flight. Integrative research into seasonal adjustments of lipid uptake regulation, muscle anabolism, and metabolic rates has provided substantial understanding as to how Catbirds cope with energetic challenges throughout their lifecycle as in Corder and colleagues [10] and DeMoranville et al., [15]. As comprehensive as these studies are, they are limited in their ability to sample birds during active spring migration and rely on capturing migrants who are at or very near their breeding grounds, when birds may be transitioning into a reproductive phenotype. We provide a critical perspective on the physiological strategies birds may employ as they simultaneously recover and prepare for the next leg of migration.

During migratory flight, birds must sustain high metabolic rates to power exercise, but upon arrival at stopover grounds, suppression of basal metabolic rate may be key to reducing maintenance costs and therefore assist rapid recovery of depleted tissues. However, Gerson and colleagues note that the foundational premise that catabolized lean tissue mass is the principal cause of reduced metabolism after flight must be thoroughly investigated [20]. Recently, Elowe et al., [17] show that catabolized protein is a critical fuel in the early stages of migratory flight in two warbler species flown under controlled conditions in wind tunnels. Furthermore, this research links high rates of lean mass catabolism and metabolic rates and suggests that lean mass may be the limiting factor for flight duration. Both the body composition and metabolic data are in congruence with field observation of the species during active migration [7, 14, 52].

Surprisingly, we did not find evidence that Catbirds increase whole-animal BMR proportionally to mass deposition during stopover recovery, as predicted. Within the long-distance migrants, we saw the opposite response which potentially indicates a substantial offset of metabolic costs typically associated with larger body masses. This finding warrants further investigation, especially in the context of stopover refueling when mass dynamics can be extreme. BMR decoupled from mass changes in this context is explained by differential contributions of organ masses to metabolism as organs may account for up to 75% of BMR while being ~ 10% of an organism’s total mass in endotherms [13, 67]. This is particularly notable in the context of migratory stopover where birds are known to have rapid adjustments of organ mass [4, 24, 57]. Griego et al., [24] demonstrates that songbirds can overcompensate intestine mass and length over the course of refueling in as little as 48 h. While that study focuses on digestive performance at the initiation of simulated stopover recovery, it sheds light on the capabilities of migratory passerines to quickly regulate digestive machinery during refueling. It may be such that songbirds are able to simultaneously reduce organ mass as they approach maximal total mass required for flight, thereby decreasing the most metabolically active tissue and suffering little energetic penalty for optimal body composition. This is in congruence with long-standing observations in shorebirds, which are hypothesized to reduce organ mass during migration to in turn lower the cost of maintenance in flight [43, 56, 65].

More recently, Vézina and colleagues [64] present evidence of differential contributions of organ mass to BMR in shorebirds under an experimentally-induced cold challenge. Cold-acclimated birds had greater total, lean, and organ mass than those in a thermal-neutral state and also exhibited an associated increase in metabolic rates. However, the authors note that *total* mass and lean mass tissues with high metabolic intensities (i.e. organ tissues, particularly kidney and liver) are both important components responsible for elevated metabolic rates. Similar findings in passerines suggest parallel strategies: seasonal adjustments in phenotypes allow volante species to cope with vastly differing challenges across life stages [42, 44, 49, 55]. We lend critical perspective as to how adjustments to body condition may not always be uniform across subpopulations with similar evolutionary history, especially when considering that individuals face differing challenges relating to migratory distance.

## Conclusion

Ultimately, an integrative understanding of the limits of phenotypic flexibility during stopover can shed substantial light on species’ resiliency. Migratory stopover refueling is a critical phase linking songbirds’ wintering and breeding grounds, with extreme significance for both individual breeding success and population stability as a whole. We present a unique approach to measuring physiological performances amongst Catbirds at stopover by relating refueling rates, body composition, and metabolism to three distinct migratory strategies. Our data reveal that long-distance fliers are able to increase refueling performance to deposit fat and lean mass without elevated metabolic costs. We strongly urge ongoing exploration of the interplay of between body condition, whole-animal and organ tissue-level performance particularly during transitory timescales which have such enormous consequence to the life cycles of billions of migratory birds in North America.

## Electronic supplementary material

Below is the link to the electronic supplementary material.

Supplementary Material 1

Supplementary Material 2

Supplementary Material 3

Supplementary Material 4

## Data Availability

All data sets used in this manuscript are hosted on a GitHub repository (https://github.com/mgriego11/Migratory-Catbird-Movement-Ecology-Data) and made available as supplementary material.
