# Supplementary material for: Divergent migratory strategies lead to variable refueling performance amongst Gray Catbirds (Dumetella carolinensis) during spring stopover in the Gulf of Mexico
Source: Mov Ecol. 2025 Oct 15;13:73. doi: 10.1186/s40462-024-00518-1 (PMC12522247; doi:10.1186/s40462-024-00518-1)
Supplement: Supplementary file 3 — Supplementary Material 3 [file 40462_2024_518_MOESM3_ESM.docx]

Divergent migratory strategies lead to variable refueling performance amongst Gray catbirds (Dumetella carolinensis) during spring stopover in the Gulf of Mexico

Michael Griego

September 2024 Publish

## This is the script used to generate plots used in this manuscript along with rudimentary data exploration.

library(ggplot2)
library(emmeans)

##Begin with analysis of [TRIG] and Body composition. This is figure 5 of the manuscript

ggplot(data = df, aes(y= trig, x= d_mass)) + geom_point() +
 geom_smooth(method=lm,se = FALSE) +
 theme_bw() +
 theme(axis.line = element_line(colour = "black"),
 panel.grid.major = element_blank(),
 panel.grid.minor = element_blank(),
 panel.border = element_blank(),
 panel.background = element_blank())

## `geom_smooth()` using formula = 'y ~ x'


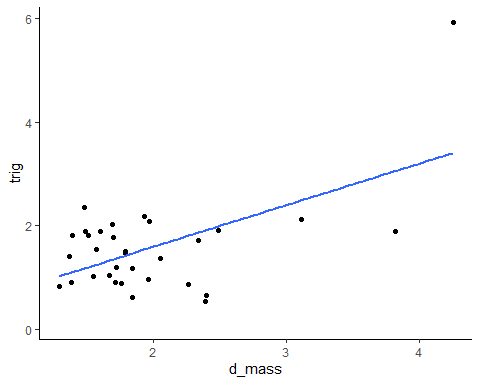


lm1<-lm(trig~d_mass, data = df)
summary(lm1)

##
## Call:
## lm(formula = trig ~ d_mass, data = df)
##
## Residuals:
## Min 1Q Median 3Q Max
## -1.3615 -0.3952 -0.1607 0.5441 2.5191
##
## Coefficients:
## Estimate Std. Error t value Pr(>|t|)
## (Intercept) -0.01276 0.44095 -0.029 0.977102
## d_mass 0.80344 0.21201 3.790 0.000678 ***
## ---
## Signif. codes: 0 '***' 0.001 '**' 0.01 '*' 0.05 '.' 0.1 ' ' 1
##
## Multiple R-squared: 0.3237, Adjusted R-squared: 0.3012
## F-statistic: 14.36 on 1 and 30 DF, p-value: 0.0006784

## NExt, we assess body condition (total, fat, and lean mass) This is figure 3 of the manuscript.

df <- df[!(is.na(df$mig_dist)), ]

ggplot(data = df, aes(x= mig_dist, y= pre_mass, na.omit = TRUE )) + geom_boxplot()+
 labs(title = "")


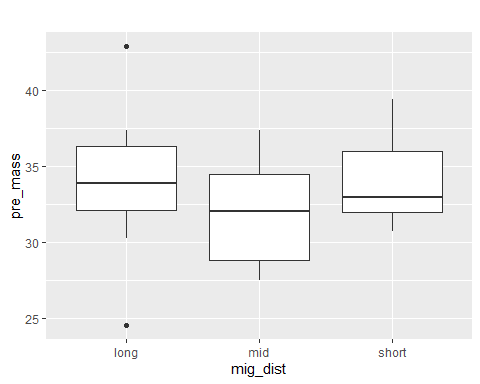


lm2 <- lm(pre_mass ~ mig_dist, data = df)
anova(lm2)

## Analysis of Variance Table
##
## Response: pre_mass
## Df Sum Sq Mean Sq F value Pr(>F)
## mig_dist 2 35.45 17.727 1.4231 0.2542
## Residuals 36 448.46 12.457

tukey2<-aov(pre_mass~mig_dist, data = df)
TukeyHSD(tukey2)

## Tukey multiple comparisons of means
## 95% family-wise confidence level
##
## Fit: aov(formula = pre_mass ~ mig_dist, data = df)
##
## $mig_dist
## diff lwr upr p adj
## mid-long -2.094091 -5.332530 1.144348 0.2669307
## short-long 0.082500 -3.526486 3.691486 0.9982803
## short-mid 2.176591 -1.832092 6.185274 0.3896662

df <- df[!(is.na(df$mig_dist)), ]

ggplot(data = df, aes(x= mig_dist, y= pre_fat, na.omit = TRUE )) + geom_boxplot()+
 labs(title = "")


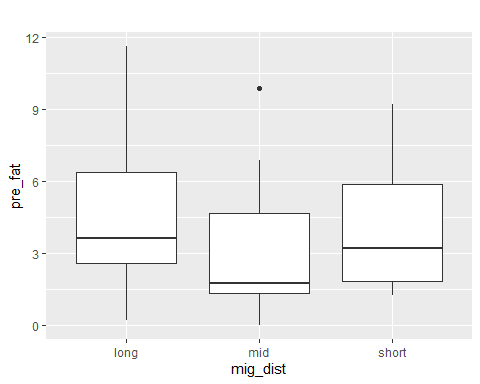


lm3<-lm(pre_fat~mig_dist, data = df)
anova(lm3)

## Analysis of Variance Table
##
## Response: pre_fat
## Df Sum Sq Mean Sq F value Pr(>F)
## mig_dist 2 10.524 5.2618 0.5999 0.5543
## Residuals 36 315.775 8.7715

tukey3<-aov(pre_lean~mig_dist, data = df)
TukeyHSD(tukey3)

## Tukey multiple comparisons of means
## 95% family-wise confidence level
##
## Fit: aov(formula = pre_lean ~ mig_dist, data = df)
##
## $mig_dist
## diff lwr upr p adj
## mid-long -1.340705 -2.9380606 0.2566515 0.1145858
## short-long -0.337750 -2.1178781 1.4423781 0.8885771
## short-mid 1.002955 -0.9743233 2.9802323 0.4379863

ggplot(data = df, aes(x= mig_dist, y= pre_lean, na.omit = TRUE )) + geom_boxplot()+
 labs(title = "")


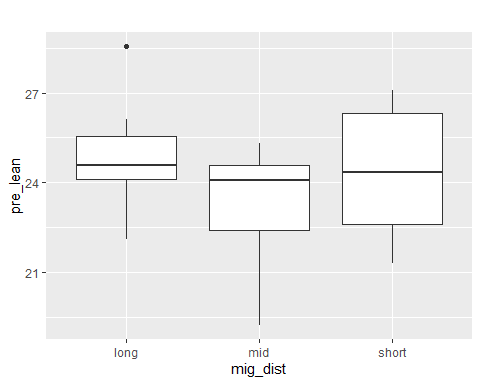


lm4<-lm(pre_lean~mig_dist, data = df)
anova(lm4)

## Analysis of Variance Table
##
## Response: pre_lean
## Df Sum Sq Mean Sq F value Pr(>F)
## mig_dist 2 12.877 6.4387 2.1244 0.1342
## Residuals 36 109.109 3.0308

tukey4<-aov(pre_lean~mig_dist, data = df)
TukeyHSD(tukey4)

## Tukey multiple comparisons of means
## 95% family-wise confidence level
##
## Fit: aov(formula = pre_lean ~ mig_dist, data = df)
##
## $mig_dist
## diff lwr upr p adj
## mid-long -1.340705 -2.9380606 0.2566515 0.1145858
## short-long -0.337750 -2.1178781 1.4423781 0.8885771
## short-mid 1.002955 -0.9743233 2.9802323 0.4379863

##Figure 4. TOtal mass of individuals vs plasma [TRIG]

ggplot(data = df, aes(y= trig, x= pre_mass)) + geom_point(aes(color = mig_dist)) +
 geom_smooth(method=lm,se = FALSE,(aes(color = mig_dist))) +
 geom_abline(aes(slope= 0.6899, intercept= 0)) +
 theme_bw() +
 theme(axis.line = element_line(colour = "black"),
 panel.grid.major = element_blank(),
 panel.grid.minor = element_blank(),
 panel.border = element_blank(),
 panel.background = element_blank())

## `geom_smooth()` using formula = 'y ~ x'

## Warning: Removed 1 rows containing non-finite values (`stat_smooth()`).

## Warning: Removed 1 rows containing missing values (`geom_point()`).


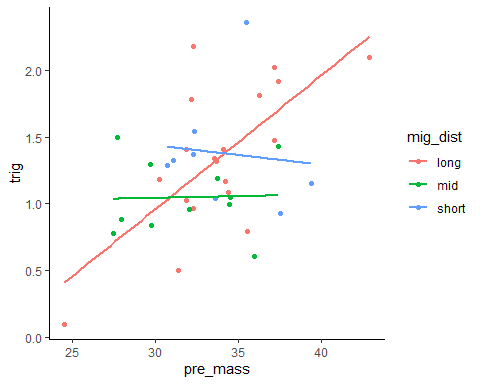


lmTrig.M<-lm(trig~pre_mass, data = df)
summary(lmTrig.M)

##
## Call:
## lm(formula = trig ~ pre_mass, data = df)
##
## Residuals:
## Min 1Q Median 3Q Max
## -0.81570 -0.23940 -0.04193 0.23696 0.97343
##
## Coefficients:
## Estimate Std. Error t value Pr(>|t|)
## (Intercept) -0.77261 0.65404 -1.181 0.24523
## pre_mass 0.06118 0.01952 3.134 0.00342 **
## ---
## Signif. codes: 0 '***' 0.001 '**' 0.01 '*' 0.05 '.' 0.1 ' ' 1
##
## Residual standard error: 0.4247 on 36 degrees of freedom
## (1 observation deleted due to missingness)
## Multiple R-squared: 0.2143, Adjusted R-squared: 0.1925
## F-statistic: 9.821 on 1 and 36 DF, p-value: 0.003424

lmTrig.M1<-lm(trig~pre_mass*mig_dist, data = df)
summary(lmTrig.M1)

##
## Call:
## lm(formula = trig ~ pre_mass * mig_dist, data = df)
##
## Residuals:
## Min 1Q Median 3Q Max
## -0.72261 -0.21205 -0.07462 0.20913 1.00022
##
## Coefficients:
## Estimate Std. Error t value Pr(>|t|)
## (Intercept) -2.04236 0.85806 -2.380 0.023429 *
## pre_mass 0.10011 0.02522 3.970 0.000381 ***
## mig_distmid 3.01027 1.42397 2.114 0.042401 *
## mig_distshort 3.91154 1.84642 2.118 0.041996 *
## pre_mass:mig_distmid -0.09761 0.04350 -2.244 0.031895 *
## pre_mass:mig_distshort -0.11458 0.05407 -2.119 0.041934 *
## ---
## Signif. codes: 0 '***' 0.001 '**' 0.01 '*' 0.05 '.' 0.1 ' ' 1
##
## Residual standard error: 0.3966 on 32 degrees of freedom
## (1 observation deleted due to missingness)
## Multiple R-squared: 0.3913, Adjusted R-squared: 0.2962
## F-statistic: 4.114 on 5 and 32 DF, p-value: 0.005357

anova(lmTrig.M1)

## Analysis of Variance Table
##
## Response: trig
## Df Sum Sq Mean Sq F value Pr(>F)
## pre_mass 1 1.7718 1.77182 11.2672 0.002047 **
## mig_dist 2 0.2829 0.14143 0.8993 0.416871
## pre_mass:mig_dist 2 1.1798 0.58992 3.7514 0.034384 *
## Residuals 32 5.0321 0.15725
## ---
## Signif. codes: 0 '***' 0.001 '**' 0.01 '*' 0.05 '.' 0.1 ' ' 1

ggplot(data = df, aes(y= trig, x= pre_fat)) + geom_point(aes(color = mig_dist)) +
 geom_smooth(method=lm,se = FALSE,(aes(color = mig_dist))) +
 theme_bw() +
 theme(axis.line = element_line(colour = "black"),
 panel.grid.major = element_blank(),
 panel.grid.minor = element_blank(),
 panel.border = element_blank(),
 panel.background = element_blank())

## `geom_smooth()` using formula = 'y ~ x'

## Warning: Removed 1 rows containing non-finite values (`stat_smooth()`).

## Warning: Removed 1 rows containing missing values (`geom_point()`).


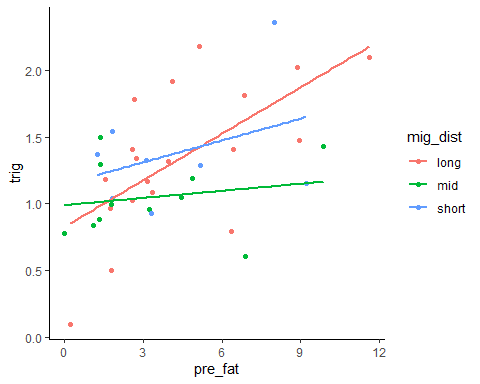


lmTrig.F<-lm(trig~pre_fat, data = df)
summary(lmTrig.F)

##
## Call:
## lm(formula = trig ~ pre_fat, data = df)
##
## Residuals:
## Min 1Q Median 3Q Max
## -0.88412 -0.18747 -0.06012 0.25558 0.82278
##
## Coefficients:
## Estimate Std. Error t value Pr(>|t|)
## (Intercept) 0.93530 0.11470 8.154 1.07e-09 ***
## pre_fat 0.08119 0.02290 3.546 0.00111 **
## ---
## Signif. codes: 0 '***' 0.001 '**' 0.01 '*' 0.05 '.' 0.1 ' ' 1
##
## Residual standard error: 0.4125 on 36 degrees of freedom
## (1 observation deleted due to missingness)
## Multiple R-squared: 0.2589, Adjusted R-squared: 0.2383
## F-statistic: 12.57 on 1 and 36 DF, p-value: 0.001107

ggplot(data = df, aes(y= trig, x= pre_lean)) + geom_point(aes(color = mig_dist)) +
 geom_smooth(method=lm,se = FALSE,(aes(color = mig_dist))) +
 geom_abline(aes(slope= 0.6899, intercept= 0)) +
 theme_bw() +
 theme(axis.line = element_line(colour = "black"),
 panel.grid.major = element_blank(),
 panel.grid.minor = element_blank(),
 panel.border = element_blank(),
 panel.background = element_blank())

## `geom_smooth()` using formula = 'y ~ x'

## Warning: Removed 1 rows containing non-finite values (`stat_smooth()`).

## Warning: Removed 1 rows containing missing values (`geom_point()`).


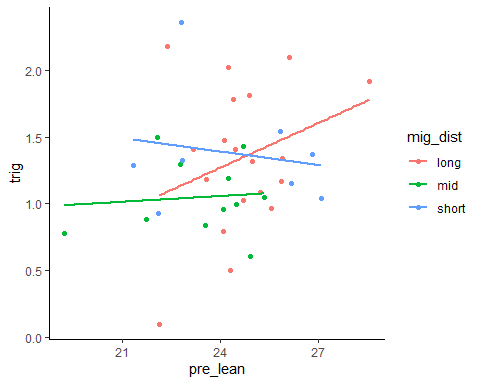


## Generate plots used in figure 6 using metabolic rate and body comp data

## First, toal mass vs vo2

ggplot(data = df, aes(y= mean_VO2, x= pre_mass)) + geom_point(aes(color = mig_dist)) +
 geom_smooth(method=lm,se = FALSE,(aes(color = mig_dist))) +
 theme_bw() +
 theme(axis.line = element_line(colour = "black"),
 panel.grid.major = element_blank(),
 panel.grid.minor = element_blank(),
 panel.border = element_blank(),
 panel.background = element_blank())

## `geom_smooth()` using formula = 'y ~ x'

## Warning: Removed 6 rows containing non-finite values (`stat_smooth()`).

## Warning: Removed 6 rows containing missing values (`geom_point()`).


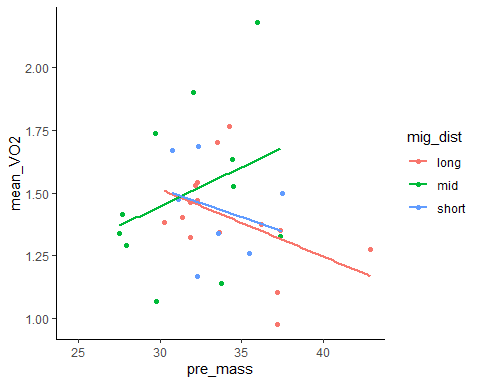


lmvo2<-lm(mean_VO2~pre_mass*mig_dist, data = df)
anova(lmvo2)

## Analysis of Variance Table
##
## Response: mean_VO2
## Df Sum Sq Mean Sq F value Pr(>F)
## pre_mass 1 0.02049 0.020488 0.3288 0.5711
## mig_dist 2 0.05396 0.026979 0.4329 0.6530
## pre_mass:mig_dist 2 0.24816 0.124078 1.9911 0.1561
## Residuals 27 1.68255 0.062317

##get slopes

lmvo2$coefficients

## (Intercept) pre_mass mig_distmid
## 2.323285446 -0.026921124 -1.811045891
## mig_distshort pre_mass:mig_distmid pre_mass:mig_distshort
## -0.133543686 0.058083224 0.004443053

ggplot(data = df, aes(y= mean_VO2, x= pre_lean)) + geom_point(aes(color = mig_dist)) +
 geom_smooth(method=lm,se = FALSE,(aes(color = mig_dist))) +
 theme_bw() +
 theme(axis.line = element_line(colour = "black"),
 panel.grid.major = element_blank(),
 panel.grid.minor = element_blank(),
 panel.border = element_blank(),
 panel.background = element_blank())

## `geom_smooth()` using formula = 'y ~ x'

## Warning: Removed 6 rows containing non-finite values (`stat_smooth()`).

## Warning: Removed 6 rows containing missing values (`geom_point()`).


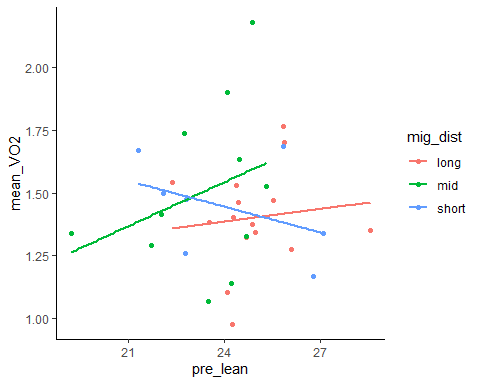


lmvo2L<-lm(mean_VO2~pre_lean*mig_dist, data = df)
anova(lmvo2L)

## Analysis of Variance Table
##
## Response: mean_VO2
## Df Sum Sq Mean Sq F value Pr(>F)
## pre_lean 1 0.00017 0.000173 0.0026 0.9595
## mig_dist 2 0.08552 0.042759 0.6506 0.5297
## pre_lean:mig_dist 2 0.14493 0.072463 1.1025 0.3465
## Residuals 27 1.77453 0.065723

lmvo2L$coefficients

## (Intercept) pre_lean mig_distmid
## 0.97827856 0.01692487 -0.84142389
## mig_distshort pre_lean:mig_distmid pre_lean:mig_distshort
## 1.29071149 0.04167477 -0.05126065
