# Supplementary material for: Divergent migratory strategies lead to variable refueling performance amongst Gray Catbirds (Dumetella carolinensis) during spring stopover in the Gulf of Mexico
Source: Mov Ecol. 2025 Oct 15;13:73. doi: 10.1186/s40462-024-00518-1 (PMC12522247; doi:10.1186/s40462-024-00518-1)
Supplement: Supplementary file 4 — Supplementary Material 4 [file 40462_2024_518_MOESM4_ESM.docx]

Divergent migratory strategies lead to variable refueling performance amongst Gray catbirds (Dumetella carolinensis) during spring stopover in the Gulf of Mexico

Michael Griego

September 2024 Publish

## This is the script used to generate Figure 1 isoplot of the manuscript. Methods are based on Ma and Bowen (2019). The following script is using a subset of data included in the manuscript for ease of demonstration and to reduce computational resources.

library(assignR)

library(terra)

## terra 1.7.39

library(rnaturalearth)

## Support for Spatial objects (`sp`) will be deprecated in {rnaturalearth} and will be removed in a future release of the package. Please use `sf` objects with {rnaturalearth}. For example: `ne_download(returnclass = 'sf')`

library(rnaturalearthdata)

##
## Attaching package: 'rnaturalearthdata'

## The following object is masked from 'package:rnaturalearth':
##
## countries110

library(ggplot2)
library(raster)

## Load the North America map data, and plot it to visualize the region.

plot(naMap)


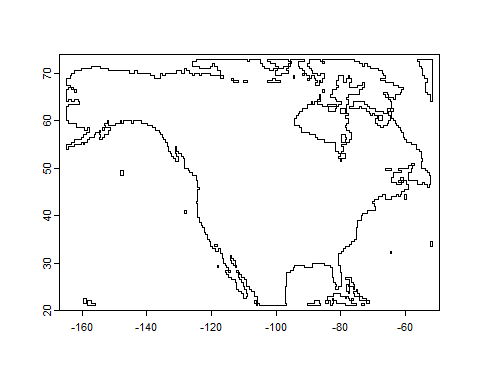
 ## Load and plot the North American d2H isoscape.

plot(d2h_lrNA)


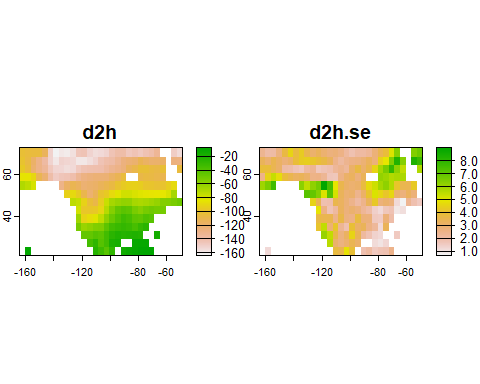


## Explore the known origin data; taxa must be biologically relevant to your focal species

names(knownOrig) # View the column names of the dataset

## [1] "sites" "samples" "sources"

## use View(knownOrig) to open the dataset in a viewer for manual inspection

## Subset the known origin data to include only ‘Passerine’ and apply the NA map as a mask.

d <- subOrigData(group = "Passerine", mask = naMap)

## 1397 samples are found from 741 sites

## Warning in refTrans(result, marker, ref_scale, niter): No calibration scale
## reported, some samples dropped from scale transformation

## 1222 samples from 727 sites in the transformed dataset


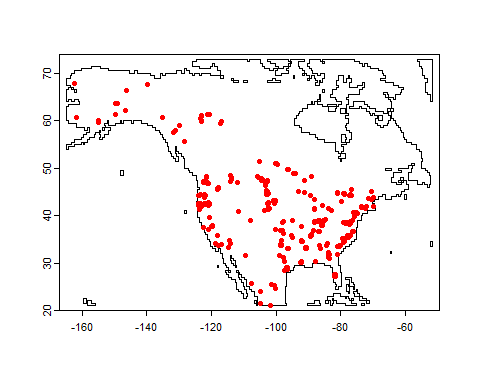


# Calculate the isoscape raster for the subset of known origin data.
r <- calRaster(known = d, isoscape = d2h_lrNA, mask = naMap)

##
##
## ---------------------------------------
## ------------------------------------------
## rescale function uses linear regression model,
## the summary of this model is:
## -------------------------------------------
## --------------------------------------
##
## Call:
## lm(formula = tissue.iso ~ isoscape.iso[, 1], weights = tissue.iso.wt)
##
## Weighted Residuals:
## Min 1Q Median 3Q Max
## -134.526 -11.582 2.049 14.409 77.840
##
## Coefficients:
## Estimate Std. Error t value Pr(>|t|)
## (Intercept) 0.47675 0.98170 0.486 0.627
## isoscape.iso[, 1] 0.84361 0.01438 58.649 <2e-16 ***
## ---
## Signif. codes: 0 '***' 0.001 '**' 0.01 '*' 0.05 '.' 0.1 ' ' 1
##
## Residual standard error: 21.98 on 1220 degrees of freedom
## Multiple R-squared: 0.7382, Adjusted R-squared: 0.738
## F-statistic: 3440 on 1 and 1220 DF, p-value: < 2.2e-16


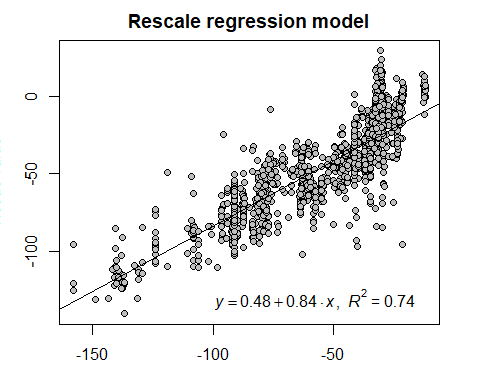

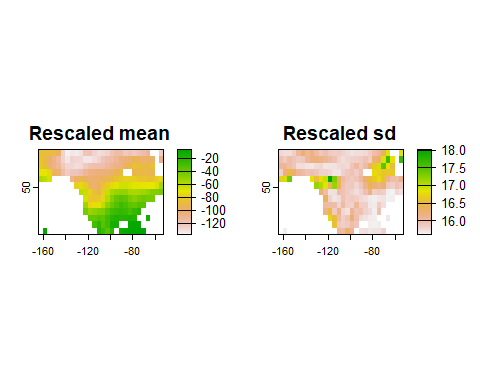


## NULL

# Plot the rescaled isoscape mean with custom colors and labels.
Isoscape1 <- plot(r$isoscape.rescale$mean,
 legend = TRUE,
 col = bpy.colors(16),
 xlab = "longitude",
 ylab = "latitude",
 legend.args = list(text = "d2H"))

## Warning in plot.window(...): "legend.args" is not a graphical parameter

## Warning in plot.xy(xy, type, ...): "legend.args" is not a graphical parameter

## Warning in title(...): "legend.args" is not a graphical parameter


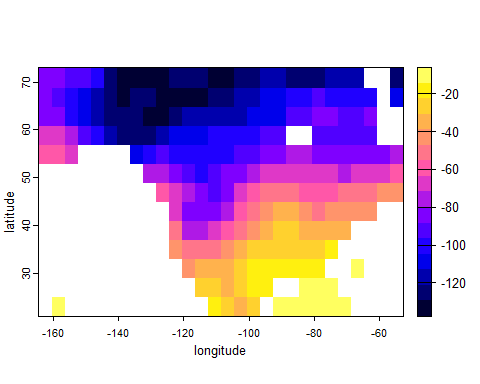
 ## Process the GRCA data

# Remove missing data from the isotope dataset.
GRCA_Isotopes_long <- na.omit(df)

# Subset the origin data for the specific taxon 'Seiurus aurocapilla'; Ovenbird chosen due to similar molt patterns during life history thus incorporating d2H at breeding grounds similar to Catbird

Ll_d <- subOrigData(taxon = "Seiurus aurocapilla", mask = naMap)

## 104 samples are found from 51 sites

## Warning in refTrans(result, marker, ref_scale, niter): No calibration scale
## reported, some samples dropped from scale transformation

## 52 samples from 43 sites in the transformed dataset


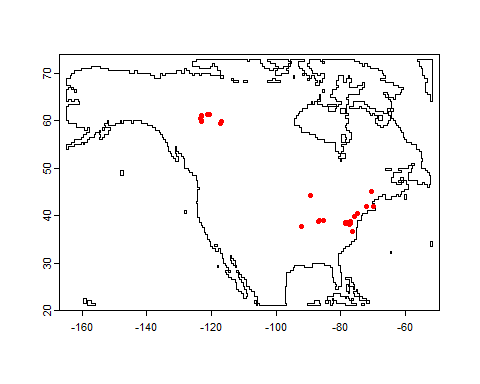


# Display the chain information from the subset data.
Ll_d$chains

## [[1]]
## [1] "OldEC.1_H_1" "EC_H_7" "EC_H_9" "VSMOW_H"

# Calculate the raster for the subset data with the isoscape and mask.
d2h_Ll <- calRaster(known = Ll_d, isoscape = d2h_lrNA, mask = naMap)

##
##
## ---------------------------------------
## ------------------------------------------
## rescale function uses linear regression model,
## the summary of this model is:
## -------------------------------------------
## --------------------------------------
##
## Call:
## lm(formula = tissue.iso ~ isoscape.iso[, 1], weights = tissue.iso.wt)
##
## Weighted Residuals:
## Min 1Q Median 3Q Max
## -21.084 -9.851 0.539 7.627 47.138
##
## Coefficients:
## Estimate Std. Error t value Pr(>|t|)
## (Intercept) -9.08028 2.92746 -3.102 0.00316 **
## isoscape.iso[, 1] 0.74304 0.02751 27.013 < 2e-16 ***
## ---
## Signif. codes: 0 '***' 0.001 '**' 0.01 '*' 0.05 '.' 0.1 ' ' 1
##
## Residual standard error: 14.74 on 50 degrees of freedom
## Multiple R-squared: 0.9359, Adjusted R-squared: 0.9346
## F-statistic: 729.7 on 1 and 50 DF, p-value: < 2.2e-16


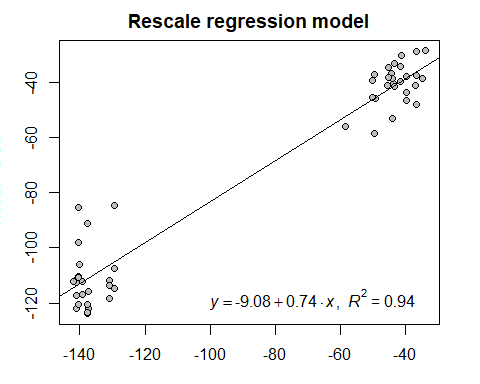

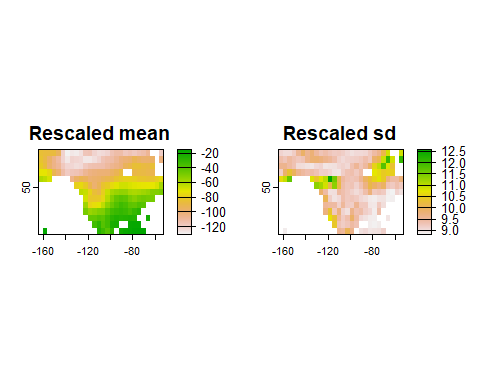


## NULL

# Calculate the probability distribution raster for the unknown GRCA isotope data.
Ll_prob_long <- pdRaster(d2h_Ll, unknown = GRCA_Isotopes_long)


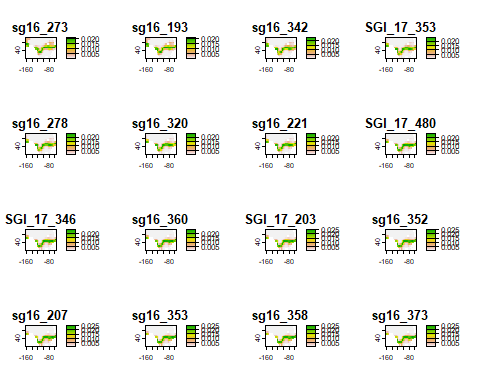


## NULL

# Increase the resolution of the raster by disaggregating with a bilinear method.
L1_prob_hi <- disagg(Ll_prob_long, fact = 20, method = "bilinear")

# Plot the quantile raster with a specified threshold.
qtlRaster(L1_prob_hi, threshold = 0.3)


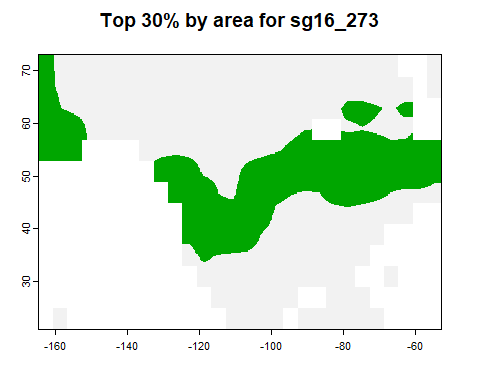

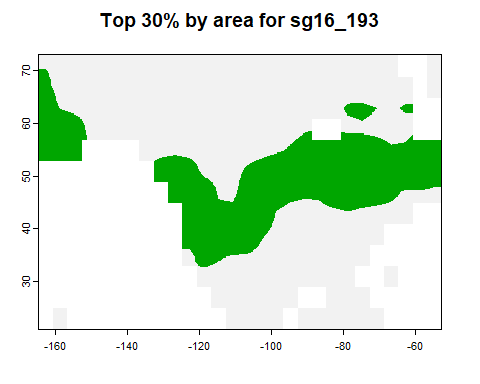

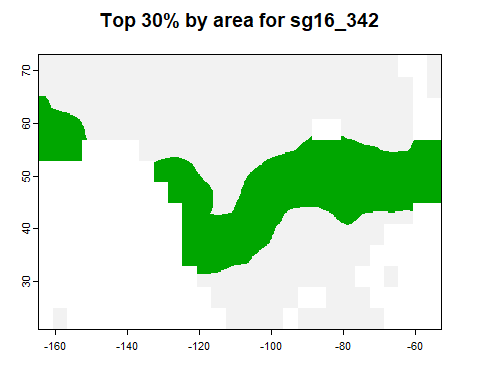

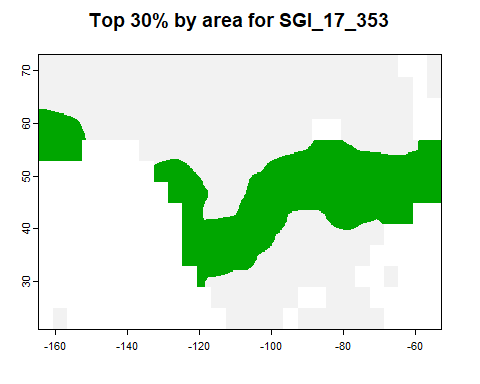

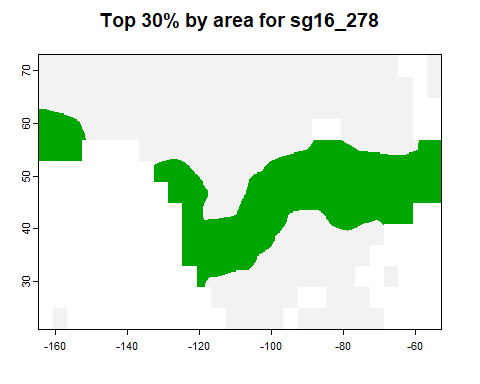

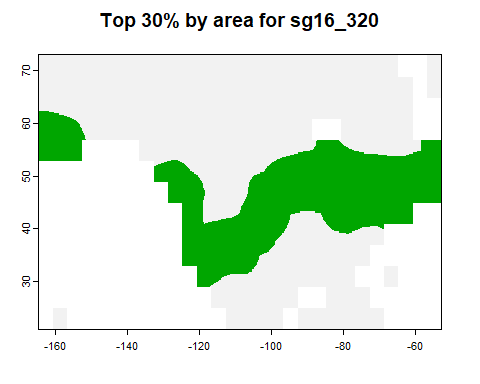

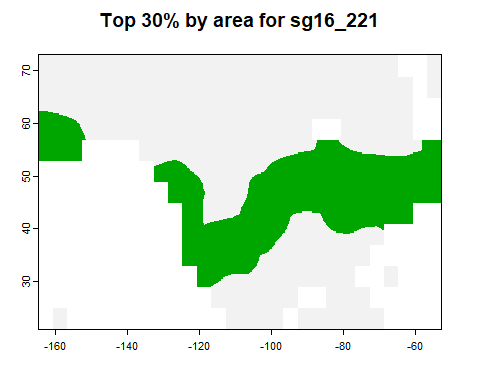

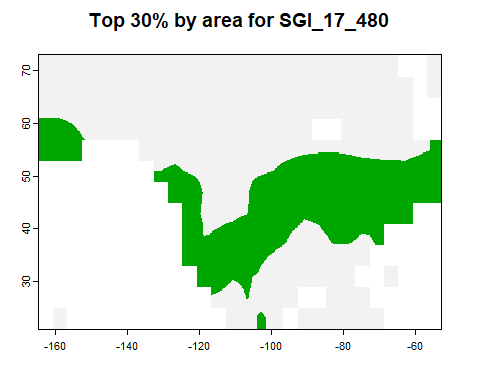

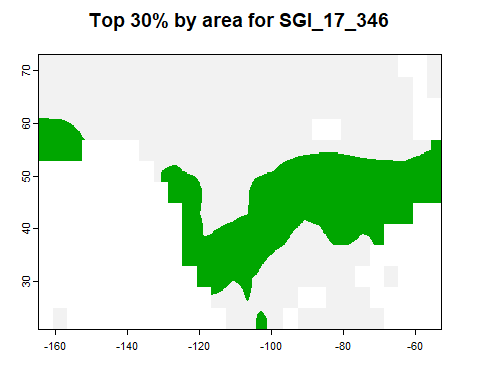

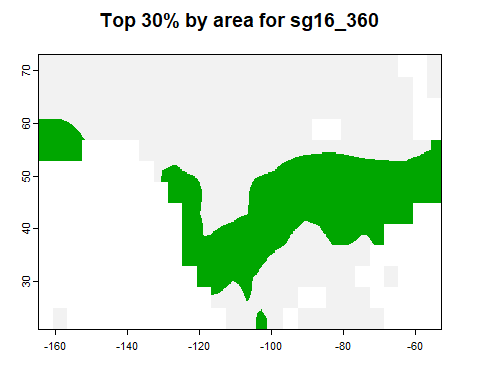

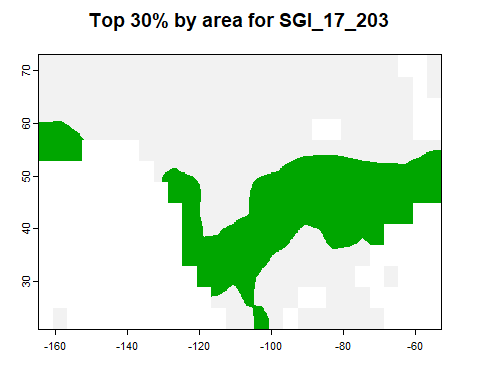

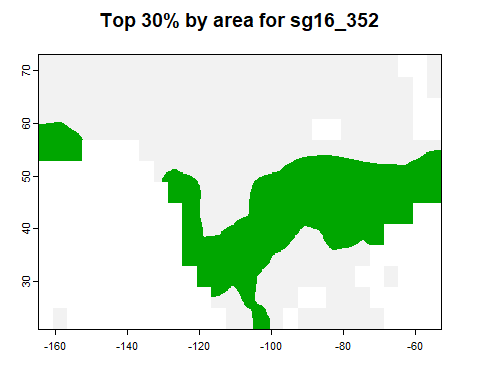

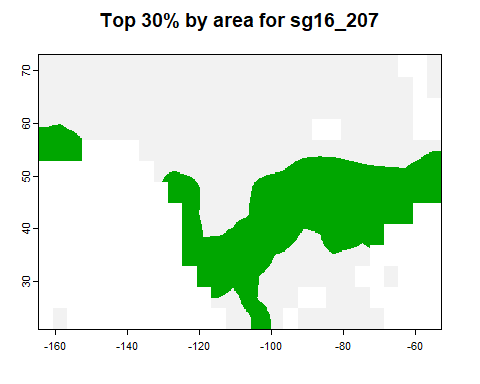

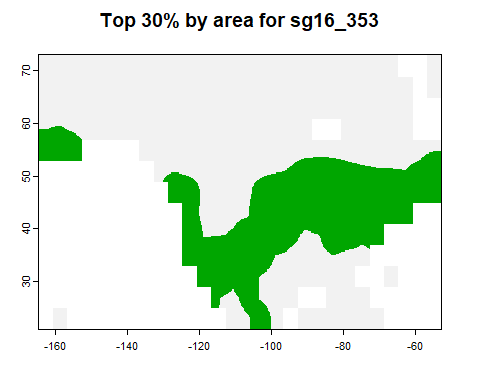

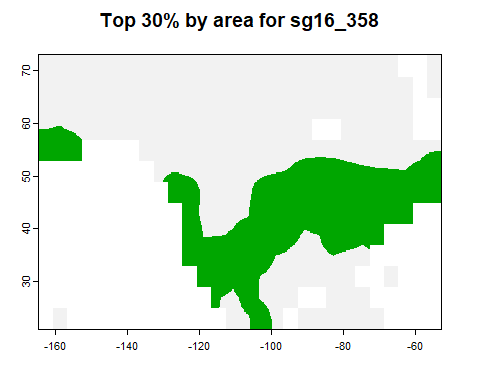

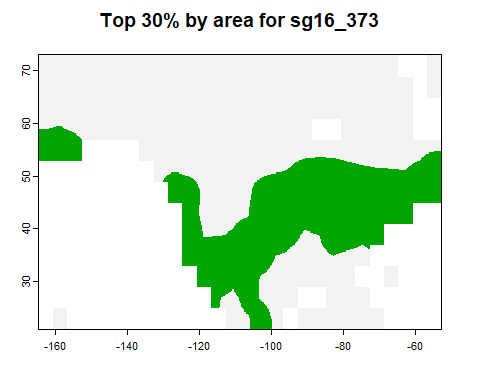

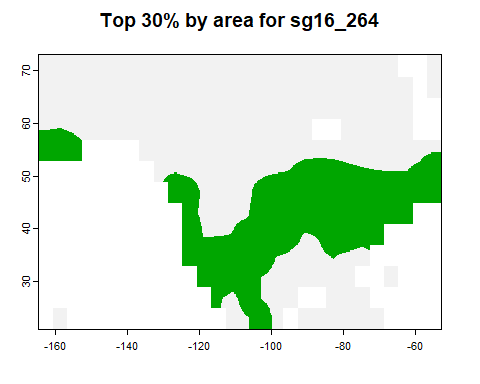

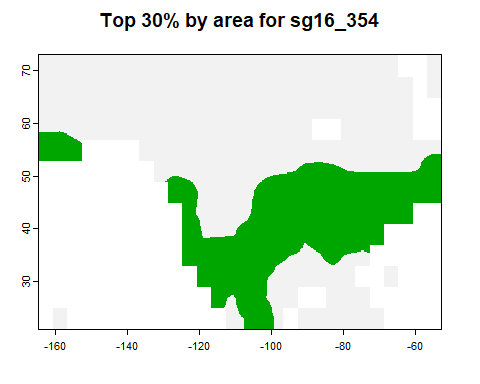

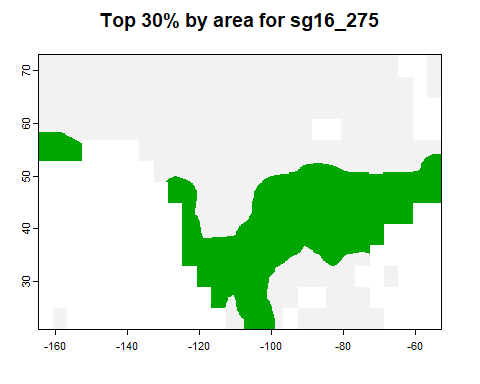

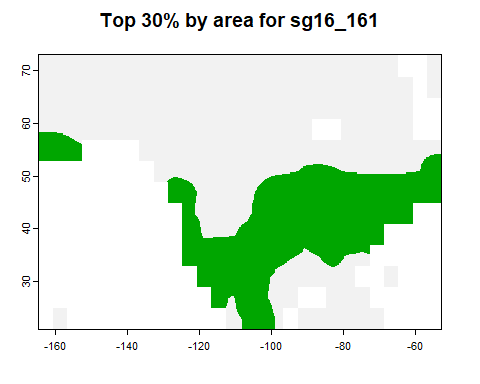


## class : SpatRaster
## dimensions : 260, 560, 20 (nrow, ncol, nlyr)
## resolution : 0.1999999, 0.1999999 (x, y)
## extent : -164.6667, -52.66672, 20.99996, 72.99993 (xmin, xmax, ymin, ymax)
## coord. ref. : lon/lat WGS 84 (EPSG:4326)
## source(s) : memory
## names : sg16_273, sg16_193, sg16_342, SGI_17_353, sg16_278, sg16_320, ...
## min values : FALSE, FALSE, FALSE, FALSE, FALSE, FALSE, ...
## max values : TRUE, TRUE, TRUE, TRUE, TRUE, TRUE, ...

# Calculate and display the joint probability.
jointP(L1_prob_hi)


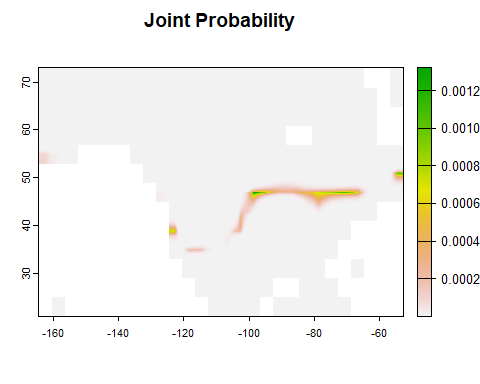


## class : SpatRaster
## dimensions : 260, 560, 1 (nrow, ncol, nlyr)
## resolution : 0.1999999, 0.1999999 (x, y)
## extent : -164.6667, -52.66672, 20.99996, 72.99993 (xmin, xmax, ymin, ymax)
## coord. ref. : lon/lat WGS 84 (EPSG:4326)
## source(s) : memory
## name : Joint_Probability
## min value : 3.668748e-247
## max value : 1.322593e-03
